# Evaluating the Socioeconomic Impact on Critical Care Delivery in Low- and Middle-Income Countries: A Prospective Observational Study From Nigeria

**DOI:** 10.1097/CCE.0000000000001337

**Published:** 2025-10-30

**Authors:** Abayomi Kolawole Ojo, Temitope Akindele Owoniya, Timilehin Mercy Jegede, Chidozie Uche Ekwem, Aghogho Ebruphyor Ajibade, Timothy Ayodele Ojo, Kwame Asante Akuamoah-Boateng

**Affiliations:** 1 Department of Anaesthesia and Intensive Care, Obafemi Awolowo University Teaching Hospitals Complex, Ile-Ife, Nigeria.; 2 Medical Laboratory Department, UNIOSUN Teaching Hospital, Osogbo, Nigeria.; 3 Department of Surgery, Division of Acute Care Surgical Services, Virginia Commonwealth University Health, Richmond, VA.

**Keywords:** critical care, critical care cost, financial burden, low-and middle-income countries, mortality, Nigeria

## Abstract

**OBJECTIVES:**

To examine the financial burden of critical care (CC), primary cost drivers, and clinical outcomes associated with CC delivery in low- and middle-income countries (LMICs).

**PERSPECTIVE:**

CC accounts for a significant cost of global health expenditure. LMICs, where the burden of critical illness is high and access remains inequitable, data on CC’s financial impact are scarce, undermining efforts to strengthen capacity, optimize delivery, and guide resource allocation.

**SETTING:**

ICU at Obafemi Awolowo University Teaching Hospital Complex, Ile-Ife, Tertiary Hospital in Nigeria.

**METHODS:**

An observational cross-sectional study was conducted. Patients were recruited via a convenient sampling method. Data were collected within the first 24 hours of admission.

**RESULTS:**

A total of 96 patients were analyzed. The average daily cost of a CC bed was ₦4,780.49 (U.S. dollar [USD] 2.99) without mechanical ventilation (MV) and ₦21,255.56 (USD 13.28) with MV. The mean expenditure within the first 24 hours of admission was ₦144,928.00 (USD 90.58). The mortality rate was 23.96%, with higher ASA scores (III and IV) and age over 40 years associated with increased costs and poorer outcomes. Higher CC costs and lower household income are strongly associated with increased mortality.

**CONCLUSIONS:**

The financial burden of CC far exceeds Nigeria’s monthly minimum wage of ₦70,000.00 (USD 43.75), highlighting the urgent need for health policy and resource allocation strategies to improve timely and equitable CC outcomes in LMICs.

**KEY FINDINGS**

**Question**: Is there a substantial financial burden in critical care (CC) delivery in Nigeria?

**Findings**: The expenditure incurred during the initial 24 hours of CC admission was equivalent to twice the monthly minimum wage in Nigeria, with essential treatments and diagnostic tests identified as the primary cost drivers.

**Meanings**: The cost of CC is high in Nigeria. Urgent restructuring of health policies, CC delivery systems, and resource allocation is needed to improve timely and equitable CC in low- and middle-income countries.

Critical care (CC) is a highly specialized care for patients with life-threatening diseases, requiring advanced care to stabilize organ dysfunction (1). A high proportion of global healthcare resources is found to be consumed by the cost of CC delivery; notably, daily mechanical ventilation (MV) alone accounts for 25.8% of these costs (2). In the United States, CC costs exceed U.S. dollar (USD) 80 billion annually, constituting approximately 1% of gross domestic product. The mean daily CC cost is three times that of the ward (3). Despite the growing need for CC post pandemic, there remain limited data in low- and middle-income countries (LMICs), especially in sub-Saharan Africa where 16% account for the global population, on CC financial burden on patients, implication to access to the care delivery, and the correlation to patient outcomes. Globally, over 100 million people are pushed into extreme poverty due to healthcare expenses, with 12.9% of households spending at least 10% of their income on medical costs and LMICs being disproportionately affected; accounting for 87% of the population facing financial hardship from out-of-pocket expenditures, leading to negative barriers such as healthcare access, delayed treatment, and increased mortality (4).

Nigeria’s growing demand for CC services is challenged by limited infrastructure and scarce financial data. With most costs borne out-of-pocket, understanding the economic burden—especially within the first 24 hours of CC admission—is vital for informed policy and resource planning. This study aims to determine the financial burden within the first 24 hours of CC admission, identify key cost drivers, and examine their relationship with clinical outcomes to support sustainable care models in LMICs.

## METHODOLOGY

This observational cross-sectional study was conducted at the ICU of the Obafemi Awolowo University Teaching Hospital Complex (OAUTHC) in Ile-Ife, Nigeria, from May 2024 to October 2024. Institutional review body, ie, the Ethics and Research Committee of OAUTHC, Ile-Ife, explicitly approved the study with protocol No. ERC/2024/04/24. Approved study title was “Evaluation of the Financial Burden of Critical Care in a Low- and Middle-Income Country” and dated April 24, 2024. The procedures of the study were conducted in accordance with the ethical standards of the responsible committee on human experimentation (institutional or regional) and with the Helsinki Declaration of 1975.

Patients were recruited using a convenience sampling based at admission to the ICU, and informed consent was obtained. Data collection occurred within the first 24 hours of admission. Data collected included demographic, admission diagnosis, number of organ failure (OF), ASA physical classification, monthly family income (MFI), ICU length of stay (LOS), mortality rate, interventions received, and direct and indirect healthcare costs. To supplement financial data, patient relatives were interviewed about out-of-pocket expenses for items purchased outside of the hospital supply chain. All costs were recorded using a conversion rate of USD 1 = ₦1600.00 (Naira), based on the approximate exchange rate during the study period. Descriptive statistics were analyzed using chi-square and bivariate analyses to explore relationships among variables such as CC costs, patient age, LOS, and ASA. A significance level of *p* value of less than 0.05 was established.

## RESULTS

### Demographics

Of the 130 patients admitted to the ICU during the study period, 96 met the inclusion criteria and provided informed consent. The cohort comprised of 37 males (38.5%) and 59 females (61.5%). The most common primary diagnosis was sepsis (30%), followed by postoperative care complications (23%), trauma (11%), post-peripartum complications (10.4%), heart failure (8%), acute respiratory failure (8%), stroke (5%), and kidney disease (4.6%).

 Referral sources included the operating room (57.3%), transfers from other hospitals or the emergency department (37.5%), and the acute care ward (5.2%). ICU LOS varied, with 48% of patients staying 1–3 days, 32% staying 4–7 days, 10% staying 8–14 days, and 9% exceeding 15 days.

Clinical characteristics, including ASA physical classification, number of OFs, and MFI, and correlation with CC cost are summarized in **Table 1**. The overall mortality rate was 23.96%. Over 50% of patients presented with two or more OFs (Table 1).

**TABLE 1.** Correlation Between Variables and Cost

Variable	Frequency, *n* (%), *n* = 96	Low Cost, *n* (%), *n* = 56	High Cost, *n* (%), *n* = 40	Average Cost, Nigerian Naira (₦)	*p*
Age group, yr					
≤ 18	14 (14.58)	5 (8.93)	3 (7.5)	2,704.24	0.004
19–39	21 (21.88)	6 (10.71)	5 (12.50)	2,965.43	
40–59	43 (44.79)	35 (62.50)	23 (57.50)	3,787.48	
≥ 60	18 (18.76)	10 (17.86)	9 (22.5)	3,576.33	
Sex					
Male	37 (38.5)	24 (42.9)	13 (32.5)	16,301.87	0.304
Female	59 (61.5)	32 (57.1)	27 (67.5)	17,134.93	
ASA					
1	13 (13.5)	11 (19.6)	2 (5.0)	7,285.33	0.001
2	22 (22.9)	18 (32.1)	4 (10.0)	10,862.93	
3	41 (42.7)	21 (37.5)	20 (50.0)	12,366.93	
4	20 (20.8)	6 (10.7)	14 (35.0)	2,921.60	
Number of organ failure					
1	24 (25)	11 (19.6)	13 (32,5)	8,361.60	0.103
2	46 (47.9)	25 (44.6)	21 (52.5)	14,466.93	
3	13 (13.5)	11 (19.6)	2 (5.0)	6,363.73	
4	13 (13.5)	9 (16.1)	4 (10.0)	4,264.53	
Monthly family income					
< 50,000	18 (18.8)	9 (16.1)	9 (22.5)	2,177.07	0.485
50–100,000	44 (45.7)	26 (46.4)	18 (45.0)	7,249.07	
100–250,000	22 (22.9)	12 (21.5)	10 (25.0)	10,723.20	
250–500,000	6 (6.3)	4 (7.1)	2 (5.0)	9,645.87	
500 to above	6 (6.3)	5 (8.9)	1 (2.5)	3,641.6	

Correlation between age, sex, ASA, number of organ failure, monthly family income, and critical care cost.

### Financial Implication

Hospital bed with MV was 5–6 times more for none MV (**Table 2**). On average, fluids, blood transfusions, imaging, central venous catheter kits, and medications had significant expense on patients as summarized in Table 2. Laboratory tests alone averaged ₦37,524.24 (USD 23.45). Overall, the average cost of CC over 24 hours was ₦144,928.00 (USD 90.58), with the average total at discharge reaching ₦388,592.74 (USD 242.7) (Table 2). Nearly half of the patients (45.8%) reported earning between ₦50,000.00 (USD 31.25) and ₦100,000.00 (USD 62.50) per month equivalent to an average daily cost.

**TABLE 2.** Critical Care Intervention

Variables	Cost (Nigerian Naira [₦]), Mean ± sd	U.S. Dollar
Bed		
Bed ± oxygen	4,780.49 ± 3,317.21	2.98
Bed + mechanical ventilation	21,255.56 ± 3,798.12	13.28
Fluid		
IV fluid	10,291.67 ± 10,510.90	6.43
Blood	29,571.43 ± 9,359.70	18.48
Radiology		
CT scan	50,000.00	31.25
Ultrasound	2,000.00 ± 1,400.00	1.25
Plain x-ray	2,957.14 ± 1,079.93	1.85
Medication		
Antibiotics	28,005.58 ± 9,630.26	17.5
Antihypertensives	28,500.15 ± 7,500.56	17.81
Analgesics	22,704.92 ± 11,346.58	14.19
Sedatives	26,266.67 ± 4,618.80	16.42
Other care		
Enteral nutrition	3,060.00 ± 1,296.93	1.91
Thrombo-embolus deterrent stockings	3,000.00	1.88
Central venous catheter kits	28,000.00	17.5
Laboratory cost		
Full blood count	3,066.67 ± 1,096.11	1.92
Packed cell volume	2,030.12 ± 401.17	1.27
Electorlytes, urea, and creatinine	5,193.75 ± 1,535.39	3.26
Blood culture	10,066.67 ± 51.64	6.29
Lactate	7,420.00 ± 2,561.60	4.64
Clotting profile	6,538.46 ± 1,059.46	4.09
Liver function test	7,442.86 ± 1,083.95	4.65
Urinalysis	2,360.00 ± 244.49	1.48
Total daily cost	144,928.00 ± 8,498.88	90.58
Overall cost	388,592.74 ± 20,9499.67	242.87

Bed occupancy and critical care intervention, e.g., drugs, fluids, imaging, laboratory tests, and others.

### Factors Associated With Financial Burden

Among the study population, patients 40–59 years old—who constituted the majority—incurred significantly higher costs, as detailed in Table 1. No statistically significant association was observed between cost and sex (*p* = 0.304), OF (*p* = 0.103), or MFI (*p* = 0.485), as shown in Table 1. ASA status III and IV were significantly associated with increased costs, both with *p* values 0.001. In terms of CC mortality, both age and ASA classifications were significantly correlated; age showed a significant association with mortality (*p* = 0.043), as did ASA III and IV (*p* = 0.005). Conversely, sex (*p* = 0.695), OF (*p* = 0.166), and MI (*p* = 0.582) were not significantly associated with CC mortality (**Table 3**). As shown in **Table 4**, higher CC costs and lower household income were significantly associated with increased mortality. Mortality rates also rose with advancing age and higher ASA classifications (ASA > 1). Conversely, higher household income was associated with lower mortality.

**TABLE 3.** Determinant of Severity of Illness and Mortality

Variable	Outcome		*p*
	Alive (*n* = 73)	Mortality (*n* = 23)	
Age group, yr			
≤ 18	12 (16.44)	2 (8.70)	0.043^a^
19–39	6 (8.21)	2 (8.69)	
40–59	41 (56.16)	15 (65.22)	
≥ 60	14 (19.19)	4 (17.39)	
Sex			
Male	24 (32.88)	13 (56.52)	0.695
Female	49 (67.12)	10 (43.48)	
ASA			
1	11 (15.07)	2 (8.70)	0.005^a^
2	19 (26.03)	3 (13.04)	
3	34 (46.58)	7 (30.43)	
4	9 (12.33)	11 (47.83)	
Number of organ failure			
1	20 (27.40)	4 (17.39)	0.166
2	40 (54.80)	6 (26.09)	
> 3	13 (17.81)	13 (56.52)	
Monthly family income			
< 50,000	7 (9.59)	3 (13.04)	0.582
50–100,000	13 (17.81)	5 (21.74)	
100–250,000	28 (38.36)	6 (26.09)	
250–500,000	22 (30.14)	6 (26.09)	
500,000 to above	3 (4.11)	3 (13.04)	

a Significant p-value.

Correlation between age, sex, ASA, number of organ failure, monthly family income, and critical care outcome.

**TABLE 4.** Outcomes Variable and Mortality

Variable	Lower–Upper	OR	*p*
Age group, yr			
≤ 18	1		
19–39	0.301–21.265	2.531	0.022^a^
40–59	1.470–154.316	15.062	0.031^a^
≥ 60	1.162–21.126	4.954	0.036^a^
Sex			
Male	1		
Female	0.391–1.991	0.883	0.764
ASA			
1	1		
2	0.006–0.434	0.049	0.006^a^
3	0.007–0.259	0.042	0.007^a^
4	0.130–1.741	0.476	0.001^a^
Monthly family income			
< 50,000	1		
50.000–100,000	0.024–1.887	3.214	0.165
101,000–250,000	0.035–1.776	1.250	0.016^a^
251,000–500,000	0.043–3.713	1.273	0.014^a^
510,000–1,000,000	1.036–5.452	0.237	0.013^a^
Cost of ICU admission			
Low cost	1		
High cost	1.060–2.475	5.160	0.001^a^
Number of organ failure			
1	1		
2	0.252–3.633	1.642	0.038^a^
3	0.207–4.789	1.407	0.009^a^
4	0.528–5.235	2.6684	0.384

OR = odd ratio.

a Significant *p* value.

Logistic regression analysis of variables associated with mortality, including age, sex, ASA classification, monthly family income, cost of admission, and number of organ failures.

## DISCUSSION

Our study provided an estimate of the financial burden associated with CC in a Nigerian tertiary hospital, and the implications of CC cost on the capacity building of CC in a LMIC. Our study found that the average daily cost of CC was ₦144,928.00 (USD 90.58), significantly exceeding the national monthly minimum wage of ₦70,000.00 (USD 43.75). Similarly, CC consumed 64.7% of hospital costs, with drugs, laboratories, and imaging as key drivers. Daily costs averaged USD 255, totaling USD 1,897 per stay—USD 509 of which were for medications. Families covered nearly 68% of expenses. Sepsis and poisoning were the top admission reasons, with an average stay of 7.8 days and a 23.9% mortality rate (5). In the Western Pacific region, Mustafa et al highlighted the high cost of CC, with daily expenses ranging from USD 300–500, with only 14% of CC patients had insurance coverage, majority of the cost contributors included monitoring, hemofiltration, and consumables, with drugs, imaging, laboratory tests, and MV identified as the most significant cost drivers (6).

### Critical Care Delivery and Financial Burden

The financial burden facing LMICs underscores the urgency of adopting more efficient and sustainable models for CC delivery. Lean methodology can reduce avoidable admissions and strengthen acute-level care, while early recognition of deterioration by frontline staff helps prevent costly escalations in care. Our data show a large proportion of CC admissions stem from the operating room, highlighting Enhanced Recovery After Surgery (ERAS) protocols as a key opportunity to reduce postoperative complications and resource use. ERAS implementation has not only reduced CC admissions postoperatively but does so without increasing morbidity, translating into substantial cost savings (pre-ERAS 65.6% vs. post-ERAS 19.4%; *p* < 0.0001) and (pre-ERAS 15% vs. post-ERAS 7.6%; *p*- 0.020) (7, 8).

Our findings align with previous research showing that sepsis is a leading cause of CC admissions and a significant cost driver. In Malawi, where sepsis mortality reaches 59.3%, outcomes are worsened by limited access to antibiotics and diagnostic tools, and families must bear the cost out of pocket, highlighting how resource scarcity directly impacts survival (9). One-year costs for severe sepsis average USD 29,238—nearly triple those of nonsevere cases—with global per-patient costs ranging from USD 1,189 to USD 99,307, reflecting the substantial economic burden on healthcare systems (10, 11).

In LMICs, out-of-pocket payment models hinder timely access to CC, often delaying ICU admission and limiting treatment. Our findings reveal that lower household income is linked to higher mortality, emphasizing the urgent need to decouple survival from financial capacity. Evidence from LMICs shows that higher CC costs are associated with prolonged ICU stays, increased complications, and high mortality—underscoring the urgent need for cost-sensitive models of care (12–14).

### Limitation

This study has several limitations that should be acknowledged. First, as a single-center study with a relatively small sample size, the generalizability of our findings to other institutions or regions is inherently limited. Second, cost structures in healthcare are highly variable—not only between hospitals but also within the same institution over time—making it difficult to extrapolate our financial data universally. Third, our methodology relied on personal interviews, receipt verification, and retrospective record reviews, which may introduce selection and reporting biases. Many patient expenses incurred outside the hospital, such as some of the laboratory and radiographic studies, had to be outsourced. Our study did not capture the indirect financial pressures that threaten the sustainability of CC delivery in LMICs, such as consumable costs, including workforce salaries. These limitations highlight the need for larger, multicenter studies across multiple LMICs with standardized data collection methods to capture the economic landscape in LMIC settings more accurately.

## CONCLUSIONS

The cost of CC in Nigeria far exceeds the national minimum wage, creating a significant obstruction to equitable access to life-saving intervention. This financial strain faced in LMICs deepens systemic health disparities in already fragile CC health systems, highlighting the unfavorable patient outcomes evident in LMICs. Addressing this challenge requires integrating cost-efficiency into system design, investing in infrastructure and the workforce, and embedding CC capacity-building care models that lead to the delivery of meaningful outcomes for all.
